# Gene knock-outs in human CD34+ hematopoietic stem and progenitor cells and in the human immune system of mice

**DOI:** 10.1371/journal.pone.0287052

**Published:** 2023-06-28

**Authors:** Daniel A. Kuppers, Jonathan Linton, Sergio Ortiz Espinosa, Kelly M. McKenna, Anthony Rongvaux, Patrick J. Paddison

**Affiliations:** 1 Human Biology Division, Fred Hutchinson Cancer Center, Seattle, Washington, United States of America; 2 Translational Science and Therapeutics Division, Program in Immunology, Fred Hutchinson Cancer Center, Seattle, Washington, United States of America; 3 Department of Immunology, University of Washington, Seattle, Washington, United States of America; Fudan University, CHINA

## Abstract

Human CD34^+^ hematopoietic stem and progenitor cells (HSPCs) are a standard source of cells for clinical HSC transplantations as well as experimental xenotransplantation to generate “humanized mice”. To further extend the range of applications of these humanized mice, we developed a protocol to efficiently edit the genomes of human CD34^+^ HSPCs before transplantation. In the past, manipulating HSPCs has been complicated by the fact that they are inherently difficult to transduce with lentivectors, and rapidly lose their stemness and engraftment potential during *in vitro* culture. However, with optimized nucleofection of sgRNA:Cas9 ribonucleoprotein complexes, we are now able to edit a candidate gene in CD34^+^ HSPCs with almost 100% efficiency, and transplant these modified cells in immunodeficient mice with high engraftment levels and multilineage hematopoietic differentiation. The result is a humanized mouse from which we knocked out a gene of interest from their human immune system.

## Introduction

CD34 expression serves as a selective marker of immature hematopoietic cells [1]. In clinical practice, CD34 is used to evaluate and ensure rapid engraftment in HSC transplants [2, 3]. CD34+ populations in bone marrow or blood samples are a hematopoietic stem/progenitor mix, of which the majority of cells are progenitors [4]. Human, donor derived CD34+ hematopoietic stem and progenitor cells (HSPCs) populations have become a standard source of cells for allogenic and autologous HSC transplantations [5, 6]. As a result, there has been intense interest in genetic manipulating CD34+ HSPCs for use in the treatment of hematopoietic-related diseases, ranging from sickle cell disease (e.g., [7, 8]) to severe combined immunodeficiency [9, 10] to HIV/AIDS [11] to cancers [12].

CD34+ HSPCs are also used as *in vitro* and *in vivo* experimental models in conjunction with functional assays (e.g., colony formation, differentiation) and xenotransplantation. For *in vivo* work, novel strains of recipient mice have been developed that better support long term human hematopoiesis and multilineage development of CD34+ cells, including B and T lymphocytes, natural killer cells, monocytes, macrophages and dendritic cells [13].

For gene manipulation experiments in HSPCs, there have been numerous attempts to optimize lentiviral (lv) gene expression platforms, including those incorporating CRISPR-Cas9- or RNAi-based elements, using alternate viral envelop proteins, promoters, viral elements etc. (e.g., [14–17]). However, in our experience such enhanced lv-vectors are still subject one or more biological limitations inherent to HSPCs, either: low transduction efficiencies, transgene silencing, and/or transduction associated toxicity.

Others have begun to develop optimized protocols for gene editing that incorporate delivery of sgRNA:Cas9 nuclease ribonucleoprotein (RNP) complexes to *ex vivo* cultured human CD34+ cells. In some cases, they have reported genomic insertion-deletion (indel) frequencies greater than 90% [18]. However, these protocols still leave room for further improvement. For example, the optimized method reported by Wu. *et al*. requires the use of non-commercially available Cas9, containing an additional NLS [18]. Meanwhile, Modarai *et al*. observed highly variable indel frequencies across patient samples when utilizing the most widely published nucleofection conditions for human CD34+ cells [19]. Lattanzi *et al*. optimized and compared the efficiency of plasmid, lentiviral vector, and RNP-based delivery into HSPCs ultimately settling on RNPs as providing the best balance between editing and cell toxicity [20]. They detected >80% Cas9 in HSPCs following nucleofection, but only averaged and indel frequency of ~30%. Shapiro *et al*. have also reported an RNP nucleofection protocol that achieves editing efficiencies similar to what we report here but requires the addition of the proprietary Alt-R Cas9 Electroporation Enhancer (IDT) to achieve equivalent levels of editing [21].

Here, we report adapting and optimizing a method of sgRNA:Cas9 RNP nucleofection we use for other primary cell systems [22] to human CD34+ cells. With this optimized protocol, we consistently achieve a greater than 95% knockout efficiency using standard commercially available reagents and reduced magnitude of donor-specific variability in indel frequency as previously reported [19]. Utilizing this protocol, we are also able to simultaneously target at least 3 different genes with minimal loss in knockout frequency. We have also optimized the protocol for transplantation of edited human CD34+ cells into humanized mice.

## Materials and methods

The protocol described in this peer-reviewed article is published on protocols.io (DOI: dx.doi.org/10.17504/protocols.io.q26g7y541gwz/v2) and is included for printing purposes as S1 File and a.docx formatted version as S2 File.

### Ethics statement

De-identified human fetal liver tissues, obtained with written informed consent from the donors, were procured by Advanced Bioscience Resources, and their use was determined as non–human subject research by the Fred Hutch Institutional Review Board (6007–827). All animal experiments were approved by the Fred Hutchinson Cancer Research Center (Fred Hutch) Institutional Animal Care and Use Committee (protocol 50941).

### CD34+ HSPC culture

The G-CSF mobilized CD34+ cells were purchased from the Fred Hutchinson Cancer Center Co-Operative Center for Excellence in Hematology. The cells were quickly thawed in a 37C water bath followed by graduated osmotic equilibration by doubling the total volume with PBS + 0.5% BSA every 2 minutes for a total of 5 doubling. Following centrifugation, the cells were cultured in Stem Span II (Stem Cell Technologies) supplemented with 100 ng/mL each of TPO, IL-6, SCF and FLT-3 ligand. For all the nucleofection optimization experiments the cells were expanded for 3 days prior to nucleofection and cultured in this media for 3 days post nucleofection.

For the erythroid differentiation test the cells were initially expanded as described above. Following nucleofection, the cells were culture as described in Uchida et al. 2018 [23], in erythroid differentiation media consisting of IMDM supplemented with 20% Knockout Serum Replacement (Thermo Fisher Scientific) 2 U/mL EPO, 10 ng/mL SCF, 1ng/mL IL-3, 1 μM dexamethasone and 1 μM estradiol for 5 days. The cells were then transitioned to Erythroid maturation media containing in IMDM supplemented with 20% Knockout Serum Replacement, 2 U/mL EPO, 10 ng/mL insulin and 0.5 mg/mL holo-transferrin for 2 days followed by flow cytometry analysis. EPO was purchased from PeproTech and all other cytokines were purchased from Shenandoah Biotechnology.

### CRISPR nucleofection

#### RNP complex formation

Prior to setting up the mixture for RNP complex formation, Cas9 was diluted to a working concentration of 50 pmol/μL and the sgRNAs pooled at concentrations between 16.7 pmol/uL and 50 pmol/μL L, depending on the number of sgRNAs in the pool. The complete P3 nucleofector solution was created by mixing the Nucleofector Solution P3 with the provided Supplement at a 4.5:1 ratio. The Nucleofector solution, Cas9 and sgRNA pool were then mixed as outlined in the tables within the detailed protocol and incubated at room temperature for 15–20 mins. S1 Table includes the sequences of all sgRNAs.

#### CD34+ cell nucleofection

For each 20 μL nucleofection, 2–4 x 10^5^ cells were aliquot into a separate 1.5 mL microcentrifuge tube and 500 μL 1x PBS added to wash. Then centrifuged for 5 minutes at 350 x g, room temperature. The liquid was then aspirated as completely as possible without disrupting the cell pellet (We typically leave up to 2μL of wash solution without issue). The cell pellet was then resuspended in 20μL of RNP complexes. All of the cell-RNP solution was then transferred to one well of a 16-well Nucleocuvette strip for each sample and nucleofected in a Nucleofector 4D X unit using Program DS-150 following the manufactures instructions. The cells were recovered in each well of the 16-well strip by adding 100μl of CD34 culture media and transferring to a 96-well plate.

### CRISPR editing analysis

Nucleofected cells were harvested at indicated timepoints and genomic DNA was extracted (MicroElute Genomic DNA Kit, Omega Bio-Tek). Genomic regions around CRISPR target sites were PCR amplified using Phusion polymerase (New England BioLabs) and primers located at least 250 bp outside of sgRNA cut sites. After size verification by agaorse gel electrophoresis, PCR products were column-purified (Monarch PCR & DNA Clean-up Kit, New England BioLabs) and submitted for Sanger sequencing (Genewiz) using unique sequencing primers. The resulting trace files for edited cells versus control cells (nucleofected with non-targeting Cas9:sgRNA RNPs) were analyzed for predicted indel composition using the Inference of CRISPR Edits (ICE) web tool [24]. Indel percentage is defined in the ICE analysis as the editing efficiency (percentage of the pool with non-wild type sequence) as determined by comparing the edited trace to the control trace. Knockout percentage represents the proportion of cells that have either a frameshift or 21+ bp indel.

### Flow cytometry

To monitor erythroid differentiation in the *in vitro* cultures cells were stained with BUV395-CD235a (BD Biosciences 563810) and APC-CD71 (BD Biosciences 341029) and analyzed on a BD Symphony. To evaluate the engraftment levels and multilineage engraftment of human hematopoietic cells in mice, blood was obtained by retro-orbital collection, and RBCs were eliminated by ammonium-chloride-potassium lysis. WBCs were analyzed by flow cytometry, following standard procedures. The following Ab clones were used (all purchased from BioLegend): anti-human Abs CD3 (HIT3a), CD11b (M1/70), CD14 (M5E2 or HCD14), CD19 (HIB19), CD33 (WM53), CD34 (581), CD45 (HI30), CD66b (G10F5) and NKp46 (9E2) and anti-mouse Ab CD45-BV605 (30-F11). Dead cells were excluded by staining with 7-aminoactinomycin D.

### MISTRG mice engraftment studies

Fetal livers were cut in small fragments and then treated for 45 min at 37°C with collagenase D (Roche; 100 ng/ml), and a cell suspension was prepared. Hematopoietic cells were enriched by density gradient centrifugation (Lymphocyte Separation Medium; MP Biomedicals), followed by positive immunomagnetic selection with anti-human CD34 microbeads (Miltenyi Biotec). Purity (>90% CD34+ cells) was confirmed by flow cytometry, and cells were frozen at −80°C in FBS containing 10% DMSO.

MISTRG mice (M-CSF^h/h^ IL-3/GM-CSF^h/h^ SIRPα^h/m^ TPO^h/h^ RAG2^−/−^ IL-2Rγ^−/−^) were previously reported [13, 25]. Newborn mice (days 1–3) were sublethally irradiated (150 cGy γ-rays in a [137Cs] irradiator), and ~10,000–12,000 edited CD34+ cells in 20 μl of PBS were injected into the liver with a 22-gauge needle (Hamilton Company), as previously described [25]. Eight to 10 weeks later, engraftment levels were measured as the percentage of human CD45+ cells among total (mouse and human combined) CD45+ cells in the blood. Mice were treated with two doses (day -4 and day -2) before analysis of either PBS or Mylotarg (gemtuzumab ozogamicin, Pfizer, 5 ug/mouse) administered intravenously by retro-orbital injection. For anesthesia, mice were placed in an anesthesia chamber for 5 minutes, with 35% displacement of air by CO2 per minute. Cervical dislocation was used as a secondary method to confirm death. No methods of analgesia were used in this study.

### Immunohistochemistry

Tissues were fixed in 10% neutral buffered formalin (SigmaAldrich) and embedded in paraffin. Sections were stained with anti-human CD33 (RBT-CD33, Bio SB) or anti-human CD163 (clone EP324, Bio SB) followed by an HRP-conjugated anti-mouse secondary antibody (Leica) and revealed with the peroxidase substrate 3, 3′-diaminobenzidine (Leica).

### Western blotting

Immunoblots were performed following standard protocols (www.cshprotocols.org). CD34+ cells were lysed in Pierce RIPA buffer (89900, Thermo Fisher) with Halt protease and phosphatase inhibitor cocktail (78440, Thermo Fisher) on ice for 15 mins. Cell lysates were quantified using the Pierce BCA Protein Assay Kit (Thermo Fisher). The Trans-Blot Turbo transfer system was used according to the manufacturer’s instructions. The following commercial antibodies were used: TP53 (Cell Signaling 48818, 1:1000), Beta-actin (Cell Signaling 4970, 1:2,000). An Odyssey infrared imaging system (LI-COR) was used to visualize blots following the manufacturer’s instructions. The Image Studio software was used to semi-quantify the blots.

## Results and discussion

### Optimization of CD34+ cell nucleofection conditions for delivery of RNA and RNPs

**Fig 1:**
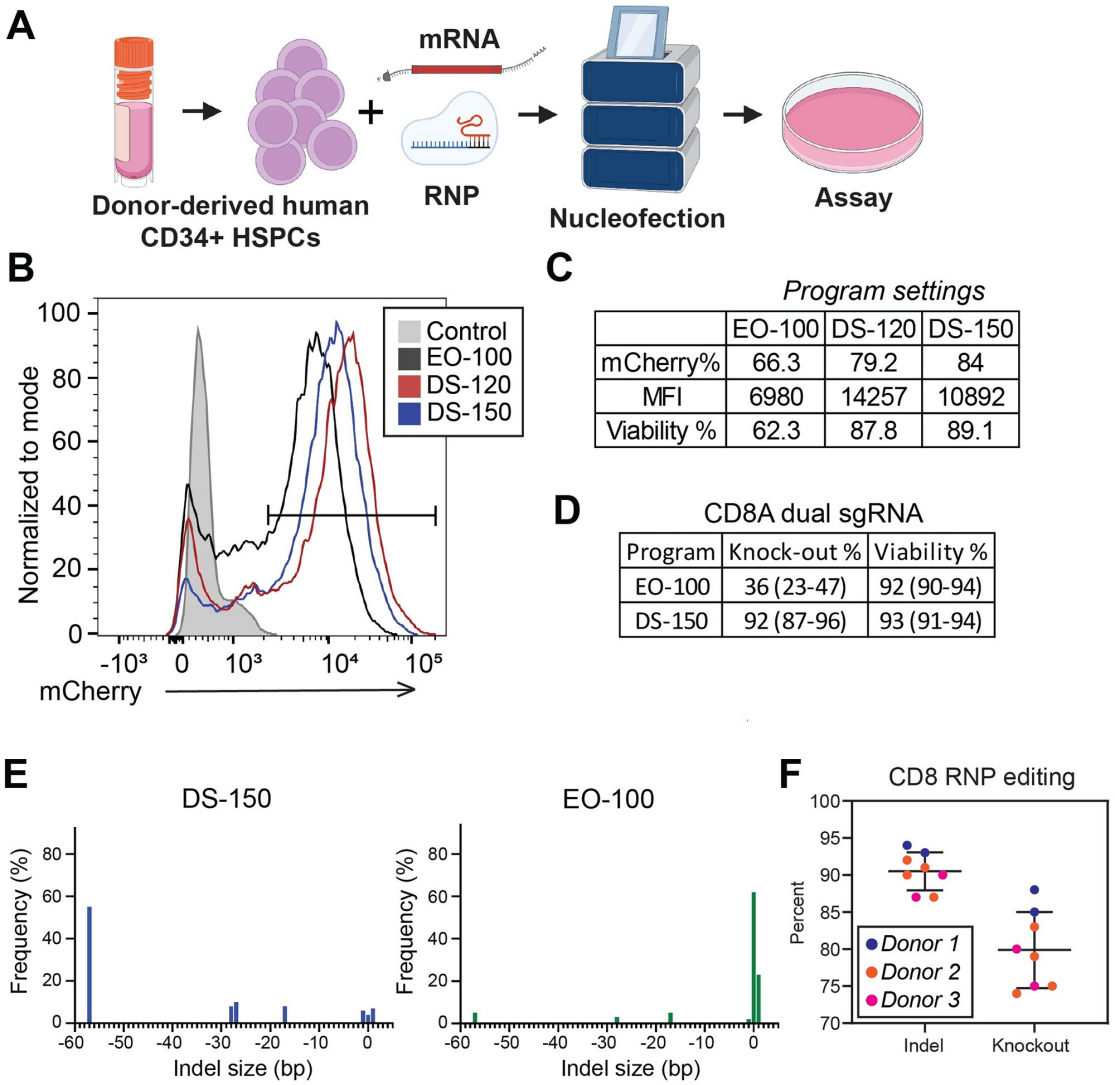
CRISPR/Cas9 RNP editing of human CD34+ hematopoietic stem and progenitor cells. (A) Overview of sgRNA:Cas9 RNP nucleofection procedure. (B) & (C) Optimization of the Lonza nucleofection program selection for CD34+ HSPCs by introducing chemically modified mCherry RNA. Levels of mCherry expression were quantified 2-days post-nucleofection by flow cytometry (n = 1). (D) Comparing the optimized and original Lonza nucleofection program by targeting CD8 in CD34+ HSPCs from three unique donors with dual sgRNAs, 50 pmol each with Cas9 in a 2:1 ratio. Three days post-nucleofection gDNA was isolated and CRISPR activity quantified by Sanger sequencing and ICE analysis (n = 3, p-value = 0.0017, the range is included in the table). (E) Representative indel size distribution plots for the data presented in D. (F) Use of the optimized protocol to target CD8 in CD34+ HSPCs from three separate human donors (n = 8). Three days post-nucleofection gDNA was isolated and CRISPR activity quantified by Sanger sequencing and ICE analysis.

Most publications employing Lonza’s 4D nucleofection technology for delivery of sgRNA:Cas9 RNPs or RNA into CD34+ cells utilize the transfection conditions outlined in the publicly available nucleofection database (knowledge.lonza.com) or very similar conditions. This standard procedure uses Kit P3 and nucleofection program EO-100 with some publications using program ER-100 [18, 19]. The reported editing efficiencies under these conditions range from 15–90%, with cell viability between 40 and 80% and significant variability in donor to donor editing efficiency [18, 19]. Based on unpublished publicly presented data, we decided to try and optimize for a more consistent editing efficiency of CD34+ cells across donors and CD34+ cell source (e.g. bone marrow, G-CSF mobilized) (Fig 1A). Utilizing chemically modified mCherry RNA (Trilink), for quantification of nucleofection efficiency, we compared the standard EO-100 program versus DS-120 and DS-150, with mock nucleofected cells as the control (Fig 1B and 1C). For both programs we measured a moderate increase in nucleofection efficiency versus the standard protocol (EO-100: 66.3%; DS-120: 79.2%; DS-150: 84.0%, n = 1)(Fig 1B and 1C), but a substantial increase in levels of mCherry expression within the cells (MFI EO-100: 6980; DS-120: 14257; DS-150: 10892, n = 1)(Fig 1B). We opted to use the DS-150 program going forward, since it resulted in a larger increase in nucleofection efficiency relative to control conditions (79.2% vs 84%), even though program DS-120 had an overall higher level of mCherry expression (MFI 14257 vs 10892). (Fig 1B and 1C). We also observe a difference in cell viability between the programs, but our overall cell viability was significantly higher than reported by others (Fig 1C) [18]. (knowledge.lonza.com).

Based on previous work in the lab to optimize sgRNA:Cas9 RNP editing in other primary cells and the work of others in CD34+ cells [18], we were able to select 50 pmol of each sgRNA and a fixed 2:1 sgRNA to Cas9 ratio for further validation of sgRNA:Cas9 RNP editing in CD34+ cells. We also standardized on using sgRNAs with 2’-O-methyl 3’ phosphorothioate modifications (in the first and last three nucleotides) from Synthego and 2xNLS SpCas9 from Aldevron. Use of chemically modified bases provides both greater sgRNA stability and decreased activation of the cellular innate immune response. To validate whether the enhanced nucleofection efficiency of program DS-150 translated to enhanced sgRNA:Cas9 RNP editing, we compared indel formation and KO efficiency of previously validated dual sgRNAs targeting CD8 and programs EO-100 and DS-150. Indel and KO efficiency was quantified as previous described [22], from Sanger sequencing data and Inference of CRISPR Edits (ICE) analysis provided by Synthego. We utilized CD34+ HSPCs from three unique donors and detected a significant increase in the average KO percentage with program DS-150 (Fig 1D)(92% vs 36%, p-value = 0.0017). Interestingly, we observed a large variation in the editing efficiency between donors, with the KO percent ranging from 23–47% with EO-100 and 87–96% with DS-150 (Fig 1D). We did not observe a difference in cell viability between the programs, as initially detected when nucleofecting mCherry (Fig 1C vs 1D). This suggests that in some circumstances a combination of individual cell culture technique and the material being nucleofected play a larger role in cell viability than the program selected. We also detected a difference in the indel pattern between the two programs (Fig 1E, S1 Fig). The low efficiency EO-100 program largely resulted in a single base-pair insertion, while the high efficiency DS-150 programs largely resulted in the genomic fragment between the two sgRNA cut sites being excised (Fig 1E). The difference in indel pattern is consistent with minimal simultaneous cutting by the two sgRNAs in the suboptimal conditions. The 1 bp insertion observed in the EO-100 condition has been linked by others to NHEJ repair of single sgRNA Cas9- induced double-stranded breaks with a 1 nt 5’ overhang [26–28].

Since we observed donor to donor variability in the editing efficiency (Fig 1D), we further tested the consistency of the protocol. We performed sgRNA:Cas9 RNP editing on CD8 using CD34+ HSPCs from three different donors, for a total of eight separate replicates where cohorts of donors were nucleofected on different days (Fig 1F). The results show a remarkable degree of consistency in the procedure for each donor with predicted indel frequencies averaging approximately 90% regardless of donor, day, or replicate and a range in KO percentage consistent with what we previously observed (9% vs 14%)(Fig 1D vs 1F). Overall, we detected consistent intra-donor editing efficiency with variable inter-donor efficiency.

### RNP nucleofection for the simultaneous targeting of 3 or more genes

**Fig 2:**
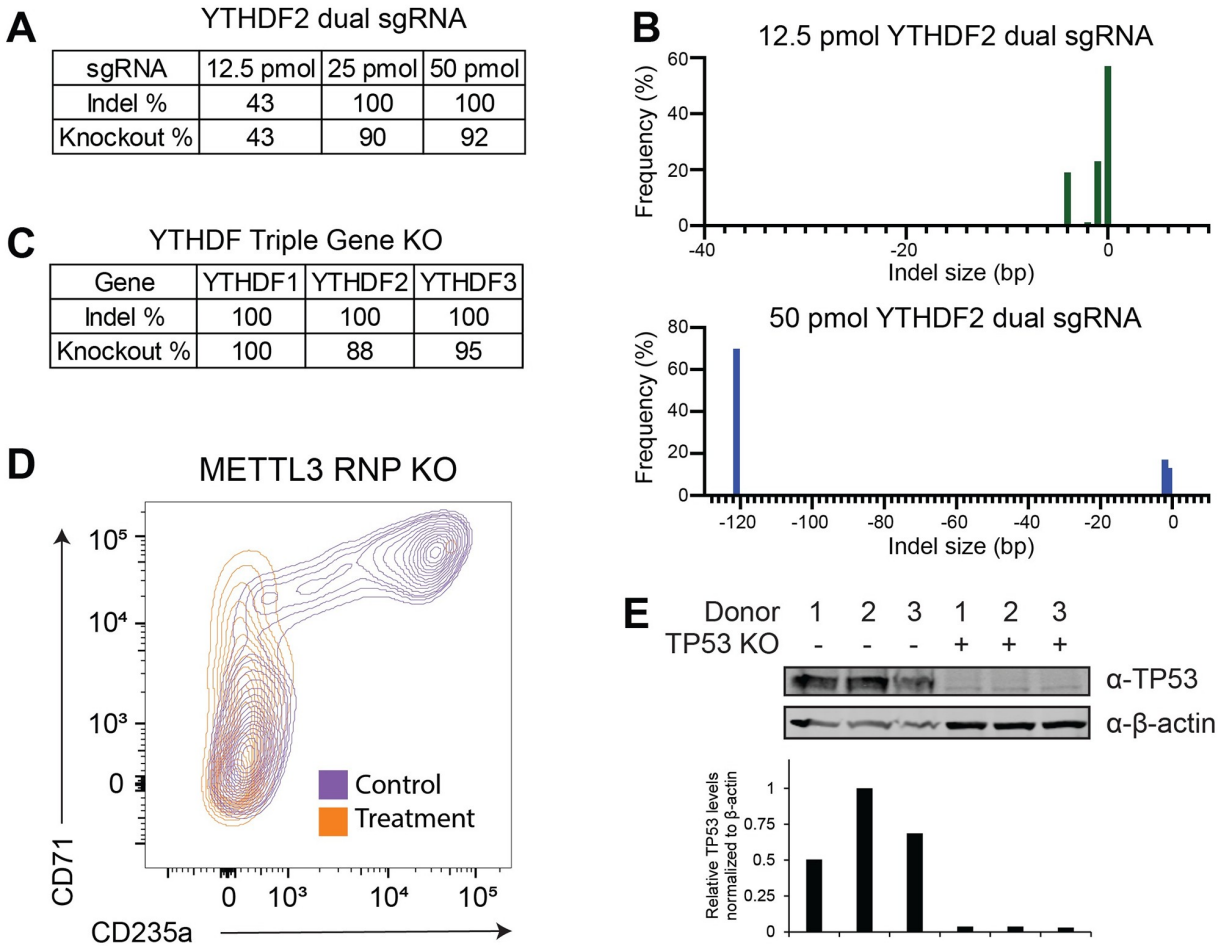
Simultaneous CRISPR/Cas9 RNP editing of multiple genes in human CD34+ hematopoietic stem and progenitor cells and in vitro biological validation of editing. (A) Optimization of input sgRNA amounts when nucleofecting dual sgRNAs for gene knockout. The listed pmole amounts were added for each sgRNA in combination with Cas9 in a 2:1 molar ratio. Three days post-nucleofection, gDNA was isolated and CRISPR activity quantified by Sanger sequencing and ICE analysis (n = 1). (B) Indel size distribution plots for the data presented in A illustrating the loss of the genomic fragment deletion with suboptimal editing efficiency. (C) Simultaneous triple gene KO by sgRNA:Cas9 RNP nucleofection. CD34+ cells were nucleofected with a pool of RNPs targeting the three YTHDF RNA binding proteins. Each pool contained 25 pmol each of 6 sgRNAs (2 sgRNAs per gene) with Cas9 in a 2:1 ratio. Three days post-nucleofection gDNA was isolated and CRISPR activity quantified by Sanger sequencing and ICE analysis. (n = 1) (D) Representative flow cytometry results of METTL3 KO by CRISPR/Cas9 RNPs recapitulating the *in vitro* erythroid differentiation phenotype previously reported by lentiviral shRNA knockdown of METTL3 [32]. Cells were assayed by FACS 7–9 days post-nucleofection. All viable cells from the nucleofection are shown. The control cells were KO for CD8 (n = 1). (E) TP53 KO by sgRNA:Cas9 RNP nucleofection. CD34+ cells from three independent donors were nucleofected with dual sgRNAs, 50 pmol each with Cas9 in a 2:1 ratio, targeting TP53. Three days post-nucleofection the cells were given 4 gray of ionizing radiation followed by isolation of protein extracts 3 hours later. TP53 protein levels were measured by Western blot with beta-actin as a loading control (n = 3). Relative levels of TP53 normalized to beta-Actin are plotted below.

The ability to simultaneously KO multiple genes has utility in a variety of situations. Among these is looking for gene dependencies when trying to identify cancer vulnerabilities [29, 30] and targeting paralogous gene families where loss of one gene may be functionally compensated for by other genes in the family [31]. The main technical limitation to target multiple genes by nucleofection is that solutions containing sgRNAs and Cas9 cannot exceed approximately 15–20% of the total volume of the 20 μl reaction. Additionally, very high KO efficiencies must be maintained for all genes, since the more genes that are simultaneously targeted, the greater the effect that small decreases in KO efficiency have on combined targeting efficiency of all the genes. We therefore opted to test the efficiency of indel formation and gene KO with decreasing sgRNA amounts of 12.5 pmol and 25 pmol, versus the 50 pmol standard per sgRNA with dual sgRNA targeting YTHDF2 and 200 thousand cells per sample in a 20 μl nucleofection reaction (Fig 2A). Maximal gene KO was detected using either 25 or 50 pmol of sgRNA with only a large reduction when using 12.5 pmol (Fig 2A). Interestingly, the reduced editing in the 12.5 pmol sample was due to near complete loss of the fragment deletion between the two sgRNAs (Fig 2B). This observation is consistent with the altered pattern of indel formation detected when comparing the low editing efficiency of EO-100 to DS-150 (Fig 1E). This suggests there is a threshold amount of RNP required to ensure the simultaneous editing necessary for a fragment deletion independent of the activity of the sgRNAs. Given these results, it may be possible to utilize a smaller amount of RNP than we observed if the sgRNAs are pre-validated to have a high KO efficiency independently.

To validate this approach, we simultaneously targeted three YTHDF m6A-binding proteins (DF1, DF2, DF3). Based on our single sgRNA results, we nucleofected 6 sgRNA pools containing 2 sgRNAs per gene and 25 pmol of each sgRNA. The pooled targeting resulted in 100% indel efficiency and 88–100% KO efficiency for all the genes (Fig 2C). When using pre-validated sgRNAs with 95% or greater KO efficiency, this pooled approach should allow the targeting of up to 6 independent genes with approximately 70% of cells being knocked out for all 6 genes and almost all cells having at least a single copy knocked out.

### *In vitro* biological validation targeting METTL3 in HSPCs

To validate the optimized RNP nucleofection conditions in a biological context, we opted to target METTL3 for KO. METTL3 is a core component of the N^6^-methyladenosine (m^6^A) mRNA methyltransferase (MTase) complex. m^6^A is an abundant RNA modification that affects the methylated transcripts in a variety of ways including altered stability and translation [33]. This regulation occurs through a host of methyltransferases, demethylases and m^6^A reader proteins, with METTL3 as one of the core components of the methyltransferase complex [34, 35] and we have previously reported that it’s knockdown (KD) by lentivirus delivered shRNAs results in a developmental block to erythropoiesis [32]. Human CD34+ HSPCs expanded for 4 days, as previously described, were nucleofected with RNPs generated from a pool of two METTL3 sgRNAs containing 50 pmol of each or the previous used dual CD8 sgRNAs as the control. Following the nucleofection the cells were placed in erythroid differentiation conditions [23]. Indel and KO frequency was quantified by ICE analysis in cells collected three days post nucleofection and erythroid differentiation by flow cytometry analysis 6-days post nucleofection (Fig 2D). We detected 91.2% biallelic gene KO and a block to erythroid differentiation consistent with METTL3’s role in erythropoiesis [32].

For further validation, we chose to look at protein loss in the CD34+ HSPCs, rather than in lineage committed progenitor cells following differentiation. Human CD34+ HSPCs expanded for 4 days were nucleofected with RNPs generated from a pool of two TP53 sgRNAs containing 50 pmol of each or dual CD8 sgRNAs as the control. The TP53 sgRNAs had been previously validated by the lab in another primary cell system [22] to have a high editing efficiency. Three days post-nucleofection, the CD34+ HSPCs were given 4 gray of ionizing radiation followed by isolation of protein extracts 3 hours later. TP53 protein levels were below the limit of detection in the TP53 KO cells as quantified by Western blot while TP53 was clearly detected in the CD8 KO control cells (Fig 2E).

### RNP nucleofected CD34+ cell transplantation into humanized mice results in greater than 95% loss of target gene expression *in vivo*

**Fig 3:**
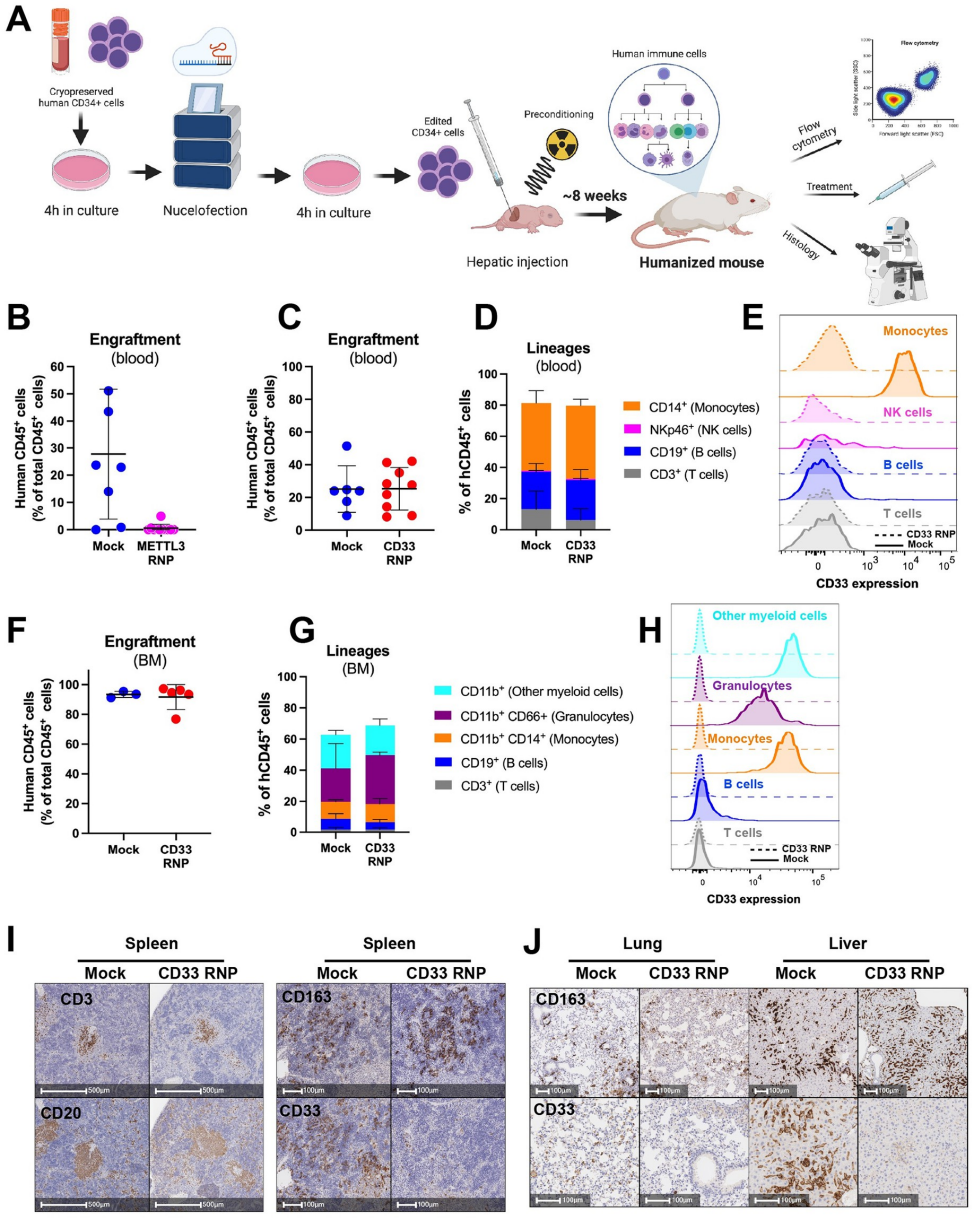
High efficiency in vivo gene KO following transplantation of CRISPR/Cas9 RNP edited human CD34+ cell into the MISTRG humanized mouse model. (A) The optimized workflow for transplantation of CRISPR/Cas9 RNP edited CD34+ human cells into MISTRG mice. (B) Frequency of human CD45+ cells in the blood of MISTRG mice 8-weeks after transplantation of fetal CD34+ cells, treated either with mock nucleofection (n = 7) or METTL3 RNP (n = 8). (C) Frequency of hCD45+ cells in the blood of MISTRG mice after transplantation of mock nucleofected (n = 6) or CD33 RNP CD34+ cells (n = 9). (D) Lineage differentiation in the blood of the same mice. (E) Expression levels of cell surface CD33 by cells of each lineage in the blood. (F) Frequency of hCD45+ cells in the BM of MISTRG mice. (G) Lineage differentiation in the BM. (H) Expression levels of cell surface CD33 by cells of each lineage in the blood. (I) IHC identifying human CD3+ T cells, CD20+ B cells and CD163+ myeloid cells, and expression of CD33 in the spleen of MISTRG mice. (J) IHC identifying human CD163+ myeloid cells and expression of CD33 in the lung and liver.

Previous efforts at gene editing in human CD34+ cells have shown both variability in editing efficiency and persistence of the edits in long-term engrafting HSCs (LT-HSC). Our optimized nucleofection protocol has addressed the first of these issues. To minimize the loss of LT-HSCs, we further optimized the protocol to minimize the time the cells are cultured *in vitro* before injection into a mouse. In brief (Fig 3A), human fetal liver-derived CD34+ HSPCs were thawed out and cultured for 4 hours in our standard HSPC expansion media, to allow them to recover from the thawing process. The cells were then nucleofected utilizing our optimized protocol and after a second recovery period of 4 hours, 10,000 cells were injected into the livers of 2-day old preconditioned MISTRG mice as previously described [13]. Initial editing was assessed by ICE analysis after *in vitro* culture of a subset of the cells for 3 days. *In vivo*, KO was assessed by flow cytometry of blood 8-weeks post-transplant.

For our first *in vivo* validation study, we again chose to target *METTL3*. A previous study established that mouse fetal liver HSPCs in which Mettl3 was conditionally deleted have dramatically lower BM engraftment than Mettl3+ cells, owing to the induction of deleterious innate immunity response [36]. To investigate whether this observation is also relevant during human hematopoiesis we utilized the optimized *in vitro* editing and transplantation protocol from above to target METTL3. Our results recapitulate the observation in mice with a near total loss of human hematopoietic cells *in vivo* in the METTL3 KO mice compared to mock nucleofected control (CD45+: 0.13% n = 11 vs. 27.81% n = 8) (Fig 3B). The KO efficiency predicted by ICE analysis was ~90%.

For our second validation study, we targeted CD33 (SIGLEC3). CD33 is a cell surface receptor for sialic acid proteins primarily expressed on myeloid cells, with KO of CD33 shown to not cause a developmental phenotype [37, 38]. CD33 expression is easily detectable by flow cytometry and immunohistochemistry (IHC) and is the target for the antibody-drug conjugate Mylotarg, which is used to treat acute myeloid leukemia [39]. *In vitro*, we detected approximately 87% KO of CD33 in the fetal liver HSPCs 3-days post nucleofection. In vivo, we found that the nucleofection and targeting procedure with a CD33-targeting RNP did not significantly affect engraftment rates in comparison to a mock nucleofection. Indeed, the percentage of human CD45+ hematopoietic cells in the blood and bone marrow (BM) of MISTRG mice was comparable in the mock nucleofected control and targeted groups (Fig 3C and 3F). Multilineage differentiation was also identical in both groups, with comparable frequencies of T and B lymphocytes, NK cells and myeloid cell subsets (Fig 3D and 3G). Remarkably, we observed total ablation of detectable CD33 protein expression from blood and BM myeloid cells (including monocytes and granulocytes) by flow cytometry (Fig 3E and 3H), demonstrating efficient knockout of the target gene. Similarly, IHC analysis of the spleen revealed that CD33 RNP nucleofection didn’t affect human hematopoietic lineage differentiation and spatial organization (including CD3+ T cells, CD20+ B cells and CD163+ myeloid cells), but it almost completely abrogated CD33 expression (Fig 3I). A similar efficiency of CD33 knockout was also observed in the lung and spleen (Fig 3J).

**Fig 4:**
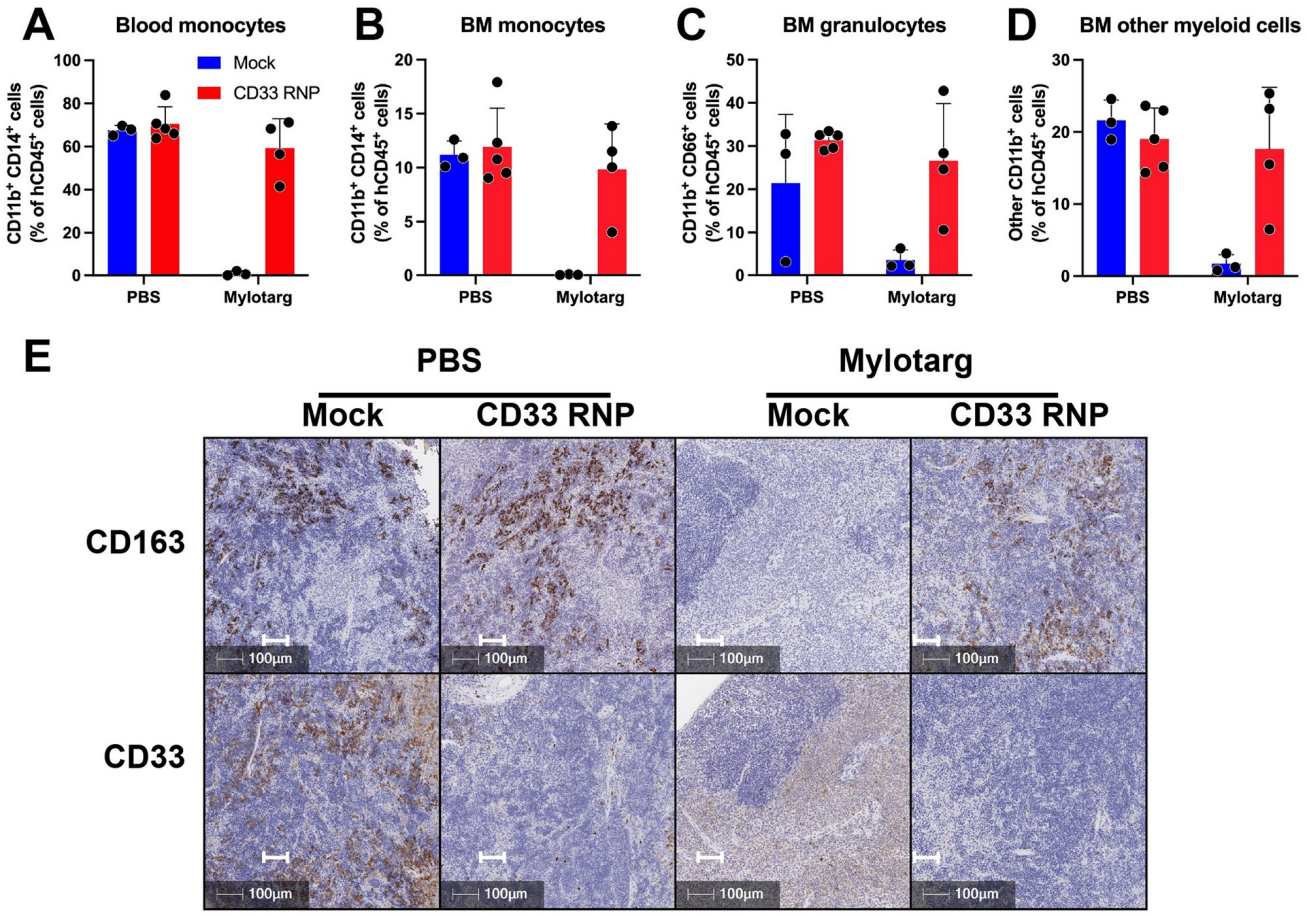
CD33 deficiency confers resistance to the CD33-targeting antibody-drug conjugate Mylotarg. Frequency of monocytes (**A**, **B**), granulocytes (**C**) and other myeloid cells (**D**) in the blood (**A**) or BM (**B-D**) of mock nucleofected control and CD33 RNP mice, after treatment with Mylotarg. (**E**) Identification of CD163+ myeloid cells and expression of CD33 in the spleen of the same mice.

Further, treatment of mice with Mylotarg showed dramatic clearance of peripheral blood and bone marrow (BM) monocytes (identified by flow cytometry as hCD45+ CD11b+ CD14+ cells), BM granulocytes (hCD45+ CD11b+ CD66+ cells), and other CD11b+ myeloid cells, which all express CD33 (Fig 4A–4D). However, CD33 KO cells were entirely resistant to Mylotarg-mediated depletion (Fig 4A–4D). This observation was corroborated by IHC analysis of the spleen, showing that CD163+ macrophages are resistant to Mylotarg in mice transplanted with edited CD34+ cells (Fig 4E). Together, these observations demonstrate that our protocol resulted in the generation of CD33 KO humanized mice.

## Conclusions

Here, we describe an improved method for highly penetrant biallelic indel formation in human CD34+ HSPCs via the use of sgRNA:Cas9 RNP nuclease complexes. This optimized protocol is higher in efficiency than previously published methods [18, 19], including those currently in clinical trials [7], and works similarly well in adult and fetal HSPCs. The two key optimizations in this method are switching to a more effective nucleofection program and the ability get high efficiency editing with reduced amounts of RNPs for any individual sgRNA. The advantages this provides are that single or multiple genes can be targeted, and that the high efficiency of targeting persists *in vivo* in engrafted cells and their progeny in the BM, peripheral blood, and organ sites. These features allow for integration of this protocol with multiple experimental paradigms, including in combination with clinically relevant agents, such as Mylotarg, as we demonstrate. Additionally, we modified the protocol to limit the time the edited CD34+ HSPCs remain in culture before transplantation into mice. We haven’t tested the impact of longer-term *in vitro* culture on the penetrance of editing and persistence of edited cells *in vivo*, so it’s presently unknown how important this variable is in obtaining the persistence of editing we detect in engrafted cells.

This method can also be used to create precise deletions using two nearby sgRNA targeting sequences, either in protein coding genes (e.g., removal of an exon) or in non-coding DNA elements such as non-coding RNAs and transcriptional enhancers/repressors [22]. Further, given the ability to scale down nucleofections, it is also amenable for small to medium-scale function genetic screens.

However, there are some limitations to this approach. First is expense. The nucleation apparatus used is more expensive than traditional electroporators and has proprietary software and settings (e.g., waveforms, capacitance, etc.) that cannot be readily reversed engineered. The chemically synthesized sgRNAs are also significantly more costly than the DNA oligos used for standard sgRNA cloning. Second, human CD34+ cells are not widely available and do require special handling and culture conditions. Third, because nuclease-based gene editing can result in large chromosomal rearrangements in a portion of cells [40], multiple controls are warranted for long-term biological assays, especially, *in vivo*. None-the-less, this should be a useful and robust method for gauging gene or genetic element requirements in pre-clinical studies of human HSPCs and their progeny both *in vitro* and *in vivo*.

## Supporting information

S1 File Protocols.io version of the detailed CD34+ HSPCs Cas9:sgRNA RNP nucleofection protocol used in this paper. (PDF)

S2 File The detailed CD34+ HSPCs Cas9:sgRNA RNP nucleofection protocol used in this paper. (DOCX)

S1 Table The sequences for all sgRNAs used in this study. (XLSX)

S2 Table The minimal supporting data for this study. (XLSX)

S1 Fig Representative Sanger sequencing traces used for ICE analysis. Sanger traces for one sgRNA from unedited input and EO-100 and DS-150 CD8 edited samples. The sgRNA cut site is denoted by the dashed red line, the sgRNA sequence highlighted in blue, and the PAM site in orange.
(TIF)

S1 Raw images (PDF)
