# Supplementary material for: Gene knock-outs in human CD34+ hematopoietic stem and progenitor cells and in the human immune system of mice
Source: PLoS One. 2023 Jun 28;18(6):e0287052. doi: 10.1371/journal.pone.0287052 (PMC10306193; doi:10.1371/journal.pone.0287052)
Supplement: S2 File — (DOCX) [file pone.0287052.s002.docx]

**CD34+ cells Cas****9:sgRNA RNP Nucleofection Protocol**

To achieve high CRISPR-Cas9 genome editing efficiencies while minimizing the length of time the cells are in ex vivo culture, this protocol employs transient nucleofection of *in vitro-*formed Cas9:sgRNA RNP complexes. This method allows for >90% indel formation in primary cell cultures in only 3 days and transplantation of cells into mice as soon as 4 hours post-nucleofection. The user can generate small indel mutations using a single sgRNA, biasing toward the generation of targeted deletions using multiple sgRNAs or the simultaneous targeting of up to 6 genes.

**Solutions and reagents:**

- RNase decontamination solution (RNaseZap, Thermo Fisher, AM9780)
- Chemically synthesized sgRNA, with 2'-O-methyl 3’phosphorothioate modifications for optimal performance. (Synthego)
- Nuclease-free 1X TE Buffer (Tris-EDTA, pH 8.0) (provided by Synthego)
- Nuclease-free water (provided by Synthego)
- P3 4D-Nucleofector Kit (Lonza, 20ul Nucleocuvette strips format, V4XP-3032 or V4SP-3096)
- sNLS-SpCas9-sNLS nuclease (Aldevron, cat. no. 9212-0.25MG)
- CD34 expansion media: StemSpan SFEM II (Stemcell Tech., 09655) with 100ng/ml TPO, SCF, IL-6, FLT-3
- gDNA column purification kit (E.Z.N.A. MicroElute Genomic DNA Kit, Omega Bio-Tek, cat. no. D3096)
- High-fidelity DNA polymerase (Phusion High-Fidelity DNA Polymerase, NEB, cat. no. M0530L); dNTPs; PCR-grade DMSO
- PCR and sequencing primers appropriate for the genomic region being targeted
- PCR column purification kit (Monarch PCR & DNA Clean-up Kit, New England BioLabs, T1030S)

**NOTE:** For ICE editing efficiency analysis after nucleofection, you must have a wild-type control Sanger sequencing trace to which your CRISPR-Cas9 targeted Sanger sequencing trace can be compared. Depending on cell availability, this sequence can be amplified from unmanipulated CD34+ cells, non-targeting or irrelevant gene targeted controls, or edited cells targeting other genes of interest.

**CD34+ cell Culture**

**NOTE:** Cells should be thawed out approximately 4 hours before nucleofection for use *in vivo*. Cells for *in vitro* experiments can be expanded for up to 4 days in CD34 expansion media prior to nucleofection, with no effect on editing efficiency or post-nucleofection cell viability.

1. Prepare an amount of CD34 expansion media appropriate for the number of cells to be thawed.
2. Equilibrate the CD34+ gradually to ensure maximum viable recovery.
   1. Prepare 35ml of room temperature thawing media, per tube of CD34+ cells, by 0.2um filtering 1x PBS with 0.5% BSA.
   2. Thaw the cells rapidly in a 37C water bath.
   3. Transfer the cells to a 50 ml conical tube.
   4. Serially dilute the thawed cells with thawing media while gently mixing the cells.
      1. Slowly add 1 ml media to the 1 ml of cells and allow 3 minutes for cells to equilibrate.
      2. Repeat this process with 2 ml of thawing media for a total volume of 4 ml.
      3. Follow the same process of doubling the volume until you have a total volume of 32 ml.
   5. Collect the cells by centrifugation for 10 minutes at 400 x g, room temperature.
   6. Aspirate the media and resuspend the cells in 1ml of thawing media.
   7. Take a small aliquot of cells to count and centrifuge the remainder at 400 x g for 5 mins.
   8. Resuspend the cells in CD34 expansion media at 2-3x10^6^/ml.
3. If expanding the cells, count the cells daily to ensure they are maintained at concentration of 2-3 x 10^6^/ml.

**CRISPR ribonucleoprotein (RNP) nucleofection**

**NOTE:** Before starting, wipe down workspace and equipment with an RNase decontamination solution (RNaseZap, Thermo Fisher) to inhibit RNases that may degrade your sgRNA and use pipet tips designated for RNA work only.

1. Reconstitute your synthetic RNA (and dilute it if desired):
   1. Briefly centrifuge your tubes or plates containing synthetic modified single guide RNA (sgRNA) oligos to ensure that the dried RNA pellet is collected at the bottom.
   2. Carefully dissolve sgRNA in the provided nuclease-free 1X TE Buffer (Tris-EDTA, pH 8.0) by adding 15μl buffer to 1.5nmol of dried sgRNA. (This will result in a final concentration of 100μM (100pmol/μl)). Flick the bottom of the tube gently so the liquid covers the bottom, then quick spin reconstituted RNA down. Dissolved RNA should be stored at -20C when not in use. Under these conditions, RNA will be stable for at least one year.

**NOTE:** It is best to store RNA in 1X TE Buffer long-term and to dilute with water only the amount for use within a week. Freeze-thawing of RNA should also be kept to a minimum.

1. Dilute Cas9: Briefly centrifuge the stock Cas9 (10 mg/mL, 61μM) to ensure that all liquid is at the bottom. To make a working stock, make a 1:1.44 Cas9:H2O dilution (e.g., 2.88μl H2O + 2μl Cas9, creating 4.88μl of 25μM Cas9). Keep diluted/undiluted Cas9 on ice.

**NOTE:** If using more than 2 sgRNAs the Cas9 needs to be diluted to 50pmol/μl.

**NOTE:** It is best to dilute Cas9 only in H_2_O or PBS (pH 7.4), not directly in Nucleofector solution. We have found that concentrated Cas9 can precipitate when added directly to some buffers. Do not freeze the diluted Cas9.

**NOTE:** We have tested several commercial sources of purified Cas9 protein and have found that Aldevron’s product provides the highest editing efficiency at the lowest dose. Other sources or in-lab purified Cas9 can also be used but may require a higher molar dose for equivalent editing efficiency.

1. Dilute and pool sgRNAs to the desired working concentration:
   1. 1 sgRNA: dilute the stock sgRNA 1:1 with nuclease-free water to a concentration of 50 pmol/μl (e.g., 2μl H2O + 2μl sgRNA, creating 4μl of 50pmol/μl sgRNA)
   2. 2 sgRNA pool: mix the two stock sgRNAs (100pmol/μl 1:1) to get a final concentration of 50 pmol/μl of each sgRNA (100pmol/μl total)
   3. 3+ sgRNA pools: mix the sgRNA stock solutions for the desired number of sgRNAs to get a final concentration of 25 pmol of each sgRNA in a total volume of 2.5μl or less (e.g., 1μl each for 6 sgRNA, creating 6μl of 16.7pmol/μl of each sgRNA and 25pmol of each sgRNA in 1.5μl).

**NOTE:** The total combined volume of sgRNA and Cas9 can't exceed 20% of the total nucleofection volume. Therefore, to create pools containing greater than 8 sgRNAs, the stock concentrations of the individual sgRNAs will need to be increased accordingly.

1. Setup the 4D nucleofector
   1. Following the manufacturer's instructions for using the Amaxa 4D Nucleofector X unit, turn on the core unit and then use the touch screen to select the appropriate options for nucleofecting a 16-well strip. Utilize Buffer P3 and program DS-150 for the CD34+ cells.
2. Assemble RNP complexes:
   1. Create 20μl of Complete Nucleofector Solution per nucleofection by mixing Nucleofector Solution P3 with the provided Supplement at a 4.5:1 ratio to (e.g., for one nucleofection, combine 16.4μl Nucleofector Solution + 3.6μl Supplement). Make a master mix for multiple nucleofections.
   2. Mix the nucleofection reaction components as listed below:

**1x Reaction for 1 or 2 sgRNA(s):**

| ***Component*** | ***1 sgRNA*** | ***2 sgRNAs*** |
| --- | --- | --- |
| Complete Nucleofector Solution | 18.0 μl | 17.0 μl |
| sgRNA(s) (**50 pmol/μl**) | 1.0 μl | 1.0 μl |
| Cas9 (**25 pmol/μl**) | 1.0 μl | 2.0 μl |

***We have found that the optimal molar ratio of sgRNA:Cas9 is 2:1.***

**1x Reaction for 3 or 6 sgRNA(s):**

| ***Component*** | ***3 sgRNAs*** | ***6 sgRNAs*** |
| --- | --- | --- |
| Complete Nucleofector Solution | 17.0 μl | 17.0 μl |
| sgRNA(s) | 1.5 μl (33.3 pmol each sgRNA/μl) | 1.5 μl (16.7 pmol each sgRNA/μl) |
| Cas9 (**50 pmol/μl**) | 1.5 μl | 1.5 μl |

***We have found that 25 pmol is the minimum amount of each individual sgRNA per nucleofection when used in pools. We have found that the optimal molar ratio of sgRNA:Cas9 is 2:1.***

- 1. Incubate the mixture at room temperature for 15-20 minutes.
  2. Store the RNPs on ice or at 4C until use (up to 24 hours).

1. Collect and prepare CD34+ cells:
   1. Label a 96-well or 24-well plate based on the number of cells to be nucleofected.

**NOTE:** Cell survival is usually highest if the cells are kept at a high density for 24 hours post-nucleofection. For CD34+ HSPCs we typically plate each nucleofection at a cell concentration of 2.0-3.0 x 10^6^/ml in CD34 expansion media.

- 1. Harvest cells normally and count. For each 20 μl nucleofection, aliquot 2-4 x 10^5^ cells into a separate 1.5 mL microcentrifuge tube and add 500 μl 1x PBS to wash. Centrifuge for 5 minutes at 350 x g, room temperature.
  2. Aspirate the liquid **as completely as possible without disrupting the cell pellet** *(We typically leave up to 2μL of media without issue*). Resuspend the cell pellet in 20μL of RNP complexes. Work quickly and avoid leaving cells in Nucleofector Solution for longer than 30 minutes total (from the time you resuspend them to the time nucleofection is complete). Avoid bubble formation.

**NOTE:** Failure to aspirate the liquid completely if you wash with 1x PBS may lead to arcing during the nucleofection, which can cause dramatic cell death and/or low transfection efficiency.

- 1. Transfer all the cell-RNP solution to one well of a 16-well Nucleocuvette strip for each sample (or individual cuvettes for 100μl nucleofections), and cover with the provided lid. Make sure there are no bubbles in your Nucleocuvette and that the sample covers the bottom of the cuvette.

1. Nucleofect cells:
   1. Insert the 16-well strip into the open Nucleofector 4D X unit. Make sure the larger gap in the 16-well strip lid is at the top of the strip, so that the yellow indicator in the X unit fits through the large gap at the top of the lid.
   2. Run the nucleofection program. After run completion, the screen should display a “+” over the wells that were successfully electroporated. Remove the strip/cuvettes from the machine.
   3. Recover the cells in each well of the 16-well strip by adding 100μl of CD34 expansion media (250μl for individual cuvettes) and gently pipetting up and down 3-4 times.

**NOTE:** Excessive pipetting can increase cell death.

- 1. Transfer the cells to the 96-well or 24-well plate prepared above.

1. Three days after nucleofection, cells may be collected for indel analysis and/or phenotypic analysis. Phenotypic analysis may need to be performed sooner, as the majority of editing happens within 24 hours and the stability of the remaining RNA/protein will determine how quickly a phenotype can be observed.

**Cell manipulation post-nucleofection**

**Freezing cells post nucleofection.**

**NOTE:** Starting at 4-hours post-nucleofection, we have found that CD34+ HSPCs can be frozen from any time point without loss of editing efficiency as measured by ICE analysis.

**NOTE:** Freezing fewer than 5 x 10^5^ cells per vial typically results in dramatically lower cell recovery post-thaw.

1. Collect and prepare CD34+ cells:
   1. Pool cells from nucleofections, with a minimum 5 x 10^5^ cells for each cryo vial, into an appropriately sized tube and add 2.5x the volume of 1x PBS to wash. Centrifuge for 5 minutes at 350 x g, room temperature.
   2. Aspirate the liquid as completely as possible without disrupting the cell pellet. Resuspend the cell pellet in 500ul of CryoStor CS10 (Sigma) if freezing less than 1 x 10^6^ cells/vial and 1ml of CryoStor CS10 if freezing up to 10 x 10^6^ cells/vial.
   3. Aliquot into cryovials and freeze using your desired method.

**Editing efficiency analysis**

1. Harvest nucleofected cells and control cells (or other suitable sources of unedited template as discussed above) collecting 50,000-100,000 cells per sample in a 1.5 mL microcentrifuge tubes. Add 500μl 1x PBS to wash. Centrifuge for 5 minutes at 350xg, to pellet the cells and resuspend in 100ul 1x PBS. The cells can either be used immediately or stored at -20°C or -80°C for several months before gDNA extraction.
2. Extract gDNA using the E.Z.N.A. MicroElute Genomic DNA Kit (Omega Bio-Tek), according to the manufacturer’s protocol.
3. Perform PCR amplification of the region around the sgRNA cut site(s):
   1. Design PCR primers at least 250-350bp outside of the sgRNA cut site. If multiple sgRNAs are being used, design primers to be approximately 250bp outside of the outermost cut sites. We usually target a 700-900bp (wild-type size) amplicon.
   2. Design at least one sequencing primer that is approximately 20-80bp inside either end of the PCR amplicon. Ensure the sequencing primer is a minimum of 100bp away from any cut site. If no suitable sequencing primer can be designed, one of the PCR primers can be substituted.
   3. Perform PCR amplification (50μl reaction) on the edited and control samples using a high-fidelity polymerase such as Phusion (ThermoFisher Scientific), following manufacturer’s suggestions for PCR conditions.

**PCR set-up for Phusion High-Fidelity DNA Polymerase**

| **Component** | **50 μl reaction** |
| --- | --- |
| 5X Phusion Buffer (HF or GC) | 10 μl |
| dNTPs (10 mM each) | 1 μl |
| Forward primer (10 μM) | 2.5 μl |
| Reverse primer (10 μM) | 2.5 μl |
| DMSO | 1.5 μl |
| Phusion polymerase | 0.5 μl |
| 100 ng gDNA template | x μl |
| H_2_O | To 50 μl total |

**Cycling conditions for Phusion High-Fidelity DNA Polymerase**

| ***Temperature*** | ***Time*** | ***Cycles*** |
| --- | --- | --- |
| 98˚C | 30 sec | 1 |
| 98˚C | 5-10 sec | 25-35 |
| Annealing Temp. | 10-30 sec |  |
| 72˚C | 15-30 sec/kb |  |
| 72˚C | 5-10 min | 1 |
| 4 ˚C | Hold | -------- |

**NOTE:** We typically include a final concentration of 3% DMSO (which aids with template denaturation) in our PCR reactions with gDNA templates.

1. Run 10 μl of the PCR reactions on an agarose gel to ensure that there is only one product of the correct size in the control sample. When using multiple sgRNAs, the edited sample may have multiple bands if your CRISPR-Cas9 RNPs created multiple deletions. If you have multiple products in the control sample, design new PCR primers.

**NOTE:** If you were able to design nested sequencing primers, we’ve had success submitting products with multiple bands, so long as the desired product constitutes the majority of the total. Alternatively, you may be able to gel purify the product if the combination of sgRNAs used produce fragments clearly distinguishable from the background in the control samples.

1. Use a column PCR purification kit (Monarch PCR & DNA Clean-up Kit) to purify the remainder of the edited and control sample PCR products, according to the manufacturer’s protocol.
2. Perform Sanger sequencing on the PCR amplicons for your edited and control samples.
3. Perform editing analysis using the Sanger trace files and the freely-available webtool “Inference of CRISPR edits” (ICE), <https://ice.synthego.com/#/>. Enter the trace files (.ab1 files) for your targeted and control samples, as well as the sequence(s) of the sgRNA(s) that you used. These tools generate a computational prediction of which overlapping DNA sequences could explain the observed trace in the targeted sample. It provides both predictions of % wild-type versus % edited sequences in the cell pool and taking into account the sequence context of the edits a predicted % knockout.

**NOTE:** We recommend ICE since it can be used for samples where up to 3 sgRNAs were used simultaneously and can detect complex edits including larger deletions spanning multiple sgRNA target sites.
