# Supplementary figures and images for: Gene knock-outs in human CD34+ hematopoietic stem and progenitor cells and in the human immune system of mice
Source: PLoS One. 2023 Jun 28;18(6):e0287052. doi: 10.1371/journal.pone.0287052 (PMC10306193; doi:10.1371/journal.pone.0287052)

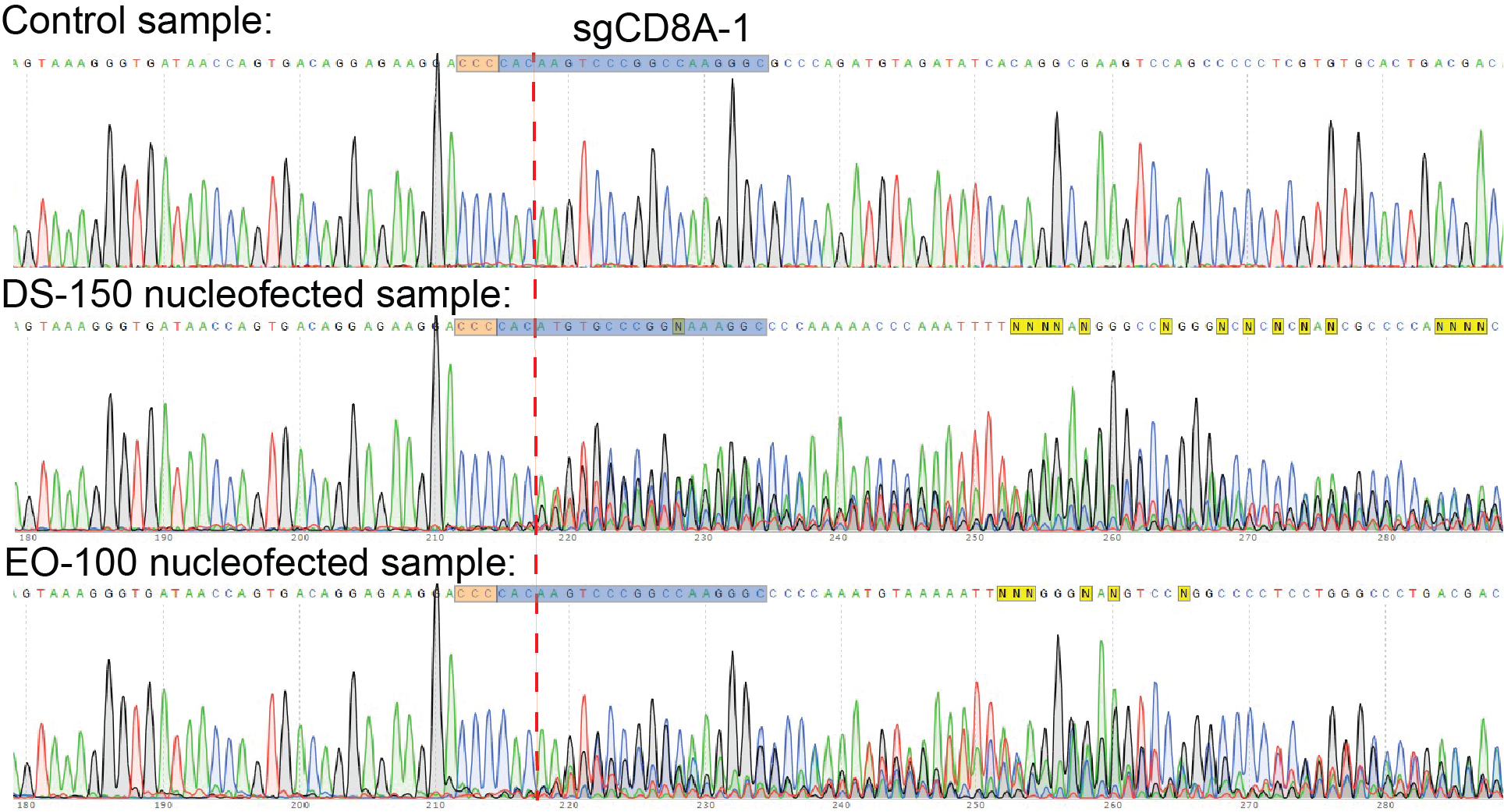

Supplement: S1 Fig — Sanger traces for one sgRNA from unedited input and EO-100 and DS-150 CD8 edited samples. The sgRNA cut site is denoted by the dashed red line, the sgRNA sequence highlighted in blue, and the PAM site in orange. (TIF) [file pone.0287052.s005.tif]

# S1\_raw\_images

Gel images supporting Fig 2E

|         |   |   |   |   |   |   |
|---------|---|---|---|---|---|---|
| Donor   | 1 | 2 | 3 | 1 | 2 | 3 |
| TP53 KO | - | - | - | + | + | + |

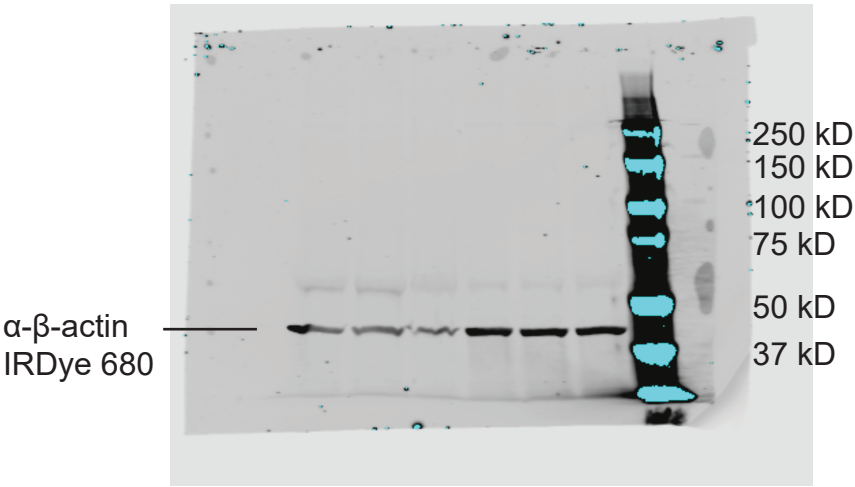

|         |   |   |   |   |   |   |
|---------|---|---|---|---|---|---|
| Donor   | 1 | 2 | 3 | 1 | 2 | 3 |
| TP53 KO | - | - | - | + | + | + |

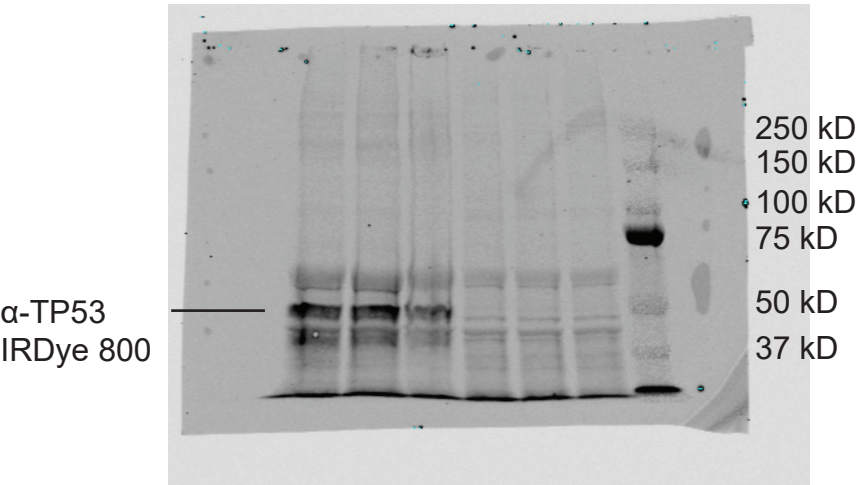

Scanned on a Li-Cor Odyssey

Supplement: S1 Raw images — (PDF) [file pone.0287052.s006.pdf]
